# The Role of *msa *in *Staphylococcus aureus *Biofilm Formation

**DOI:** 10.1186/1471-2180-8-221

**Published:** 2008-12-16

**Authors:** Karthik Sambanthamoorthy, Antony Schwartz, Vijayaraj Nagarajan, Mohamed O Elasri

**Affiliations:** 1 Department of Biological Sciences, The University of Southern Mississippi, Hattiesburg, Mississippi, 39406-0001, USA

## Abstract

**Background:**

*Staphylococcus aureus *is an important pathogen that forms biofilms. The global regulator *sarA *is essential for biofilm formation. Since the modulator of *sarA *(*msa*) is required for full expression of *sarA *and regulates several virulence factors, we examined the capacity of the *msa *mutant to form biofilm.

**Results:**

We found that mutation of *msa *results in reduced expression of *sarA *in biofilm and that the *msa *mutant formed a weak and unstable biofilm. The *msa *mutant is able to adhere to surfaces and begins to form biofilm but fails to mature indicating that the defect of the *msa *mutant biofilm is in the accumulation stage but not in primary adhesion.

**Conclusion:**

The *msa *gene plays an important role in biofilm development which is likely due to its role in modulating the expression of *sarA*. This finding is significant because it identifies a new gene that plays a role in the development of biofilm.

## Background

*Staphylococcus aureus *is *a *gram-positive pathogen that causes potentially life threatening nosocomial- and community-acquired infections, such as osteomyelitis and endocarditis. An important characteristic of *S. aureus *is its ability to form a biofilm, a characteristic associated with several diseases [1]. Bacteria in biofilms are encased in a polysaccharide glycocalyx [2], which provides them with protection against host defenses and antimicrobial drugs [2]. Staphylococcal biofilms form in two distinct stages: (1) primary adhesion to surfaces by means of adhesins or cell wall components, and (2) accumulation of multilayered clusters of cells via the production of a polysaccharide [3]. Cells are also able to detach from the biofilm and disperse to distant sites for colonization or infection.

The staphylococcal accessory regulator *sarA *is a major global regulator that is essential for biofilm formation both *in vitro *and *in vivo *[4-6]. Additionally, O'Neill *et al*.[7] showed that *sarA *is essential for biofilm formation in both MRSA and MSSA. However the mechanism of *sarA *regulation of biofilm is not yet understood. Previously we identified the *msa *gene as a positive modulator of *sarA *[8]. We also showed that mutation of *msa *resulted in differential expression of several virulence factors [8]. These findings prompted the present study, focused on the role of *msa *in biofilm formation. We show here that *msa *indeed modulates the accumulation of biofilm in *S. aureus*.

## Results and discussion

### The *msa *mutant has a weak biofilm defect

Prior studies have shown that the modulator of SarA gene (*msa*) is required for full expression of SarA, which in turn is essential for biofilm formation [4,6,8]. To examine the role of *msa *in biofilm formation, we generated an *msa *mutant in the methicillin resistant *S. aureus *(MRSA) strain COL. *S. aureus *COL was chosen for study because it forms a biofilm *in vitro *and is virulent in animal models of endocarditis [9,10]. We confirmed the *msa *mutation, and its effect on *sarA*, by measuring transcription levels of *msa *and *sarA *in the wild-type COL strain, the *msa *mutant, and the complemented *msa *mutant by real-time quantitative PCR (RT-qPCR). As expected, the *msa *mutant showed no detectable expression of *msa *in planktonic cultures or biofilm (Table 1). Additionally, transcription of *sarA *was reduced at least five-fold in these cultures in both planktonic cultures and biofilm (Table 1). These results are consistent with finding from our previous study [8] and show that *msa *is a positive modulator of *sarA *during planktonic growth as well as biofilm in strain COL.

**Table 1:** Relative expression of *msa *and *sarA *in the *msa *mutant.

		**Biofilm**		**Planktonic**					
			
		***msa *vs. COL**	**Compl. vs. COL**	**msa vs. COL**			**Compl. vs. COL**		
				
**Gene**	**Function**	**mature (12 hrs)**		**mid**	**late**	**post**	**mid**	**late**	**post**
*msa*	modulator of *sarA*	0.002	1.15	< 0.001	< 0.001	< 0.001	0.95	0.57	0.88
*sarA*	staphylococcal accessory regulator	0.30	0.77	0.22	0.21	0.12	0.76	0.78	0.48

Gene expression of *msa *and *sarA *in the *msa *mutant or the complemented mutant (Compl.) relative to wild-type (COL). Values represent the mean ratio of three independent experiments. Expression measurements were done in biofilm and three planktonic growth phases (mid-exponential, late-exponential and post-exponential).

**Figure 1:**
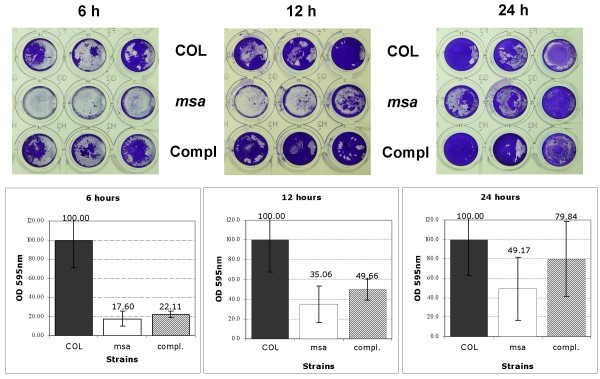
**Biofilm formation in the *msa *mutant in microtiter plates**. The wild type strain COL, the *msa *mutant and the complemented *msa *mutant were grown in TSB supplemented with NaCl and glucose. Cultures were incubated for 6, 12 and 24 hours in the wells of microtiter plates with pre-coating with plasma proteins. Biofilm was quantitated by staining with crystal violet and elution with ethanol as described in text. All values have been normalized to wild type levels which were arbitrarily set as 100%.

The biofilm forming capacity of the wild-type COL strain, the *msa *mutant, and the complemented *msa *mutant were examined by microtiter plate assay. Biofilm formation was observed in at 6, 12, and 24 h post-inoculation in the wild-type COL strain and the complemented *msa *mutant microtiter plates (Fig. 1). Conversely, there was no evidence of biofilm formation for the *msa *mutant at 6 or 12 h post-inoculation. At 24 hours post-inoculation, the *msa *mutant formed a biofilm that appeared similar to that of the wild-type COL strain (Fig. 1). These results were reproduced at least three times and were confirmed in subsequent experiments, suggesting that the *msa *mutant has a defect in biofilm growth under steady-state conditions.

**Figure 2:**
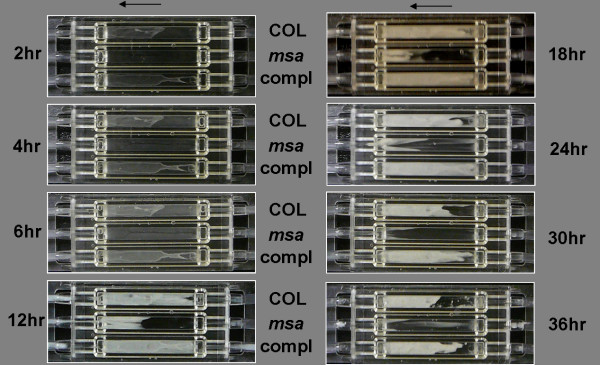
**Biofilm formation in the *msa *mutant in flow cells**. The wild type strain COL, the *msa *mutant and the complemented *msa *mutant were used to inoculate flow cells. TSB supplemented with NaCl and glucose was provided at a flow rate of 0.5 ml/minute. Biofilm formation was monitored for 36 hours. Arrow indicates the direction of flow of medium.

To further examine this phenotype, we used flow cells to test the ability of the *msa *mutant to form biofilm under shear forces. Three flow cells coated with human plasma were each inoculated with the wild-type COL strain, the *msa *mutant, and the complemented *msa *mutant, and then monitored continuously for biofilm formation while media flowed through the chamber. Wild-type COL strain and the complemented *msa *mutant formed robust and mature biofilms by 12 hours post-inoculation (Fig. 2). The *msa *mutant, however, failed to form a robust and uniform biofilm within the flow cell in the first 12 hours (Fig. 2). The gross morphology of the partial biofilm formed by the *msa *mutant was similar to those formed by the wild type strain and the complemented mutant (Fig. 2). However, while the biofilms formed by the wild-type and the complemented *msa *mutant persisted for up to 36 h before sloughing off, the biofilm formed by the *msa *mutant rapidly disintegrated (Fig. 2). These results were confirmed in three independent experiments, indicating that the *msa *mutant is defective in its ability to form mature biofilms.

Since expression of *sarA *is essential to biofilm formation, we wanted to examine the expression of *sarA *in cells forming a biofilm. We harvested biofilm from flow cells inoculated with *msa *mutant and wild-type COL at 24 h post-inoculation and measured the expression of *sarA *by RT-qPCR. We found that *sarA *expression levels were significantly reduced in the *msa *mutant, suggesting that *msa *is required for full expression of *sarA *in biofilm (Table 1). It is possible that the weak biofilm formation phenotype of the *msa *mutant could be due to a reduction in *sarA *expression.

**Figure 3:**
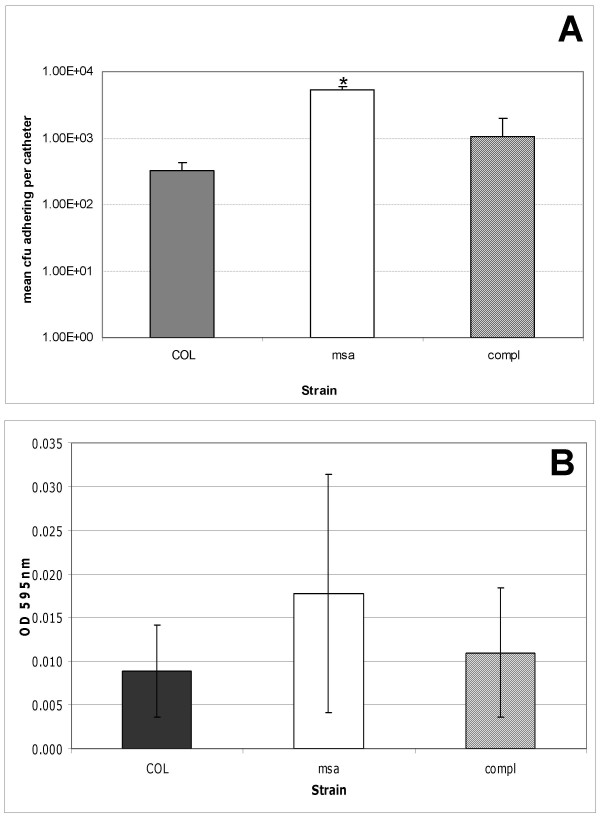
**Initial adherence assays**. A. Catheter adherence assay. Standardized overnight cultures of the wild type strain COL, the *msa *mutant and the complemented *msa *mutant were incubated with catheters at 37°C for 30 minutes. Results represent the mean ± SEM of three independent experiments. Student's paired t test was used to compare the msa mutant and the complemented msa mutant to the wild type strain (*denotes statistical significance of P < 0.05). B. Standardized overnight cultures of the wild type strain COL, the *msa *mutant and the complemented *msa *mutant were incubated for 1 hour at 37°C in plasma-coated microtitre wells. Adherent cells were then fixed with ethanol and then stained with Crystal Violet. Ethanol was then used to elute the wells and absorbance was measured. Results represent the mean ± SEM from three independent experiments.

Biofilm formation is a complex process that generally involves three stages: (1) primary adhesion to surfaces, (2) accumulation of multilayered clusters of cells, and (3) detachment. We carried out experiments to determine which stage of biofilm formation is disrupted in the *msa *mutant. Using two different adherence assays, we measured the ability of cells to attach to surfaces in the presence of host proteins by coating with human plasma and in their absence by not coating with plasma (Fig. 3). We found that the *msa *mutant had no defect in initial adherence to surfaces. In fact, the *msa *mutant adhered to surfaces significantly better than the wild-type COL strain especially in the catheter assay where no plasma was used (Fig. 3A). The complemented *msa *mutant showed a level of adhesion to surfaces that was similar to wild-type (Fig. 3). These results indicated that primary binding to surfaces was not responsible for the biofilm formation defect in the *msa *mutant. It was important to test initial adherence to surfaces with or without plasma coating because binding of host proteins is a major contributor to primary adhesion. In this case, however, we found that it does not play a role in the biofilm phenotype of the *msa *mutant.

A recent study by O'Neill et al. [11] showed that in addition to primary adherence, fibronectin binding contributes to intercellular accumulation in biofilm. We examined the ability of the *msa *mutant to bind the immobilized ligands by coating microtitre wells with fibronectin or fibrinogen and compared the capacity of the wild type COL strain, the *msa *mutant and the complemented mutant to bind these host proteins. We found that the wild type strain and the complemented mutant bind both fibronectin and fibrinogen, however, the *msa *mutant binds fibronectin but not fibrinogen (data not shown). In an effort to explain these results, we examined the expression of fibronectin-binding protein A (*fnbA*) and clumping factor (*clfA*) in biofilm (Table 2). Consistent with the binding assays, there was no significant difference in expression levels of *fnbA *between the three strains, while the expression of *clfA *in the *msa *mutant was significantly reduced in biofilm. This indicated that the lack of fibrinogen binding by the *msa *mutant is primarily due to lack of expression of ClfA.

**Table 2:** Relative expression of biofilm-related genes.

		**Biofilm**	
		
**Gene**	**Function**	**msa vs. COL**	**compl. vs. COL**
*fnbA*	fibronectin-binding protein	0.79	0.83
*clfA*	clumping factor A	0.16	0.68
*atl*	bifunctional autolysin	0.35	0.88
*alsS*	α-acetolactate synthase	9.77	1.51
*arcA*	arginine deiminase	< 0.001	0.56
*icaA*	intercellular adhesin	0.72	2.06
*spxA*	transcriptional-regulator	0.72	1.16
*tcaR*	transcription regulator	1.12	0.76

Relative expression of several biofilm-related genes in the *msa *mutant or the complemented mutant (compl.) relative to wild-type (COL) during biofilm growth. Values represent the mean ratio of three independent experiments. Genes with significant changes (twofold or higher) in expression are highlighted.

There are many other genes known to be involved in biofilm formation. The major autolysin, *atl*, has been shown to promote adherence of bacterial cells to solid surfaces [12,13]. The Atl homolog of *S. epidermidis*, AtlE, has also been shown to play an important role in primary attachment to polystyrene surfaces [14]. Gene expression studies using RT-qPCR in the *msa *mutant revealed that *atl *levels were significantly reduced in biofilm (Table 2). Therefore, our studies with *S. aureus *strain COL *msa *mutant have demonstrated that primary adhesion to surfaces with or without a plasma coat does not require full expression of the major autolysin Atl. This is consistent with previous findings in which enhanced biofilm formation occurred in the absence of the major autolysin Atl [15]. Collectively, these results suggest that the strain COL *msa *mutant is not defective in primary adhesion to surfaces, but that the defect manifests in the accumulation stage of biofilm formation.

### *msa *mutant is defective in the accumulation stage of biofilm formation

**Figure 4:**
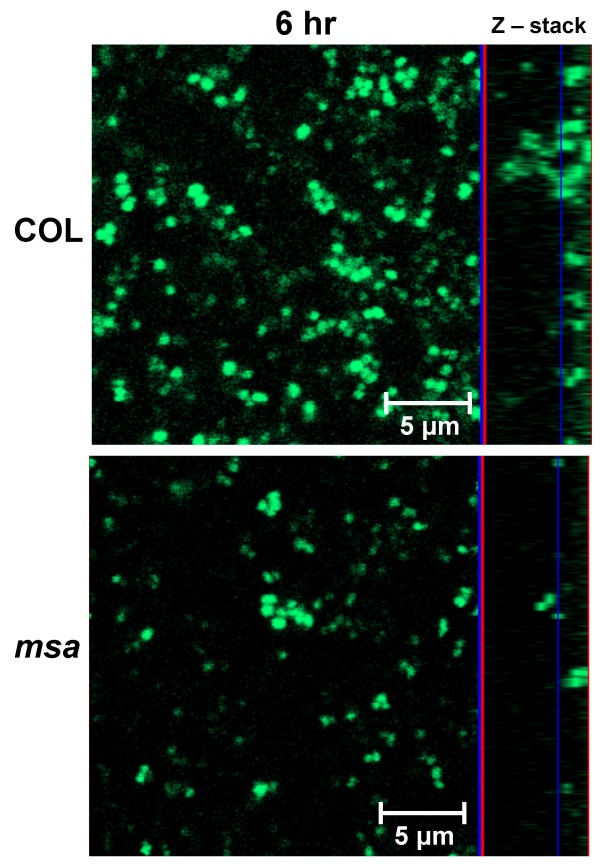
**Confocal microscopy images of biofilm**. The *msa *mutant and the wild type strain COL were imaged 6 hours post inoculation of flow cells. The panels on the left are an overlay of multiple slices, and the side frames of the panels on the right show the z-stack showing the thickness and the architecture of the biofilm. The line in the z-stack indicates the level at which the photograph of the x-y plane was taken. Photographs were taken at a magnification of ×600.

We investigated the possibility that mutation of the *msa *gene causes a growth defect that could explain a weak biofilm. We measured growth rates of the wild-type strain, the *msa *mutant, and the complemented *msa *mutant in planktonic cultures in TSB and found no significant difference between the *msa *mutant and the wild-type strain (data not shown). This was important to verify in order to eliminate the possibility of a growth defect caused by mutation of *msa*. We then measured the rate of accumulation of cells within the flow cell system for wild-type and *msa *mutant strains. In order to monitor cell deposition, we introduced into our strains plasmid pSB2019 (a kind gift from Dr. Phillip J. Hill; [16], which carries the constitutively expressed Gfp3), and used confocal microscopy to monitor biofilm formation at regular intervals. Consistent with our previous observations, there was no significant difference in initial adherence to the surface or formation of microcolonies between the *msa *mutant and the wild-type. However, the wild-type strain formed a thick biofilm as early as four hours after inoculation, while the *msa *mutant failed to develop a multilayered biofilm in the first six hours (Fig. 4). We observed a specific absence of biofilm "towers" in the *msa *mutant biofilms, as compared to the wild-type biofilms, further suggesting that the defect in biofilm formation in the *msa *mutant occurs in the accumulation stage.

To further characterize the biofilm formation defect in cells lacking *msa*, we used RT-qPCR to analyze the expression of *icaA *(*ica *operon), *arcA *(arginine deiminase), *tcaR *(transcription regulator), *atlA *(autolysin), *alsS *(alpha-acetolactate synthase), *spxA *(transcriptional regulator), genes that are reported to be involved in biofilm development (Table 2). Some of these selected loci are regulated by *sarA *(*alsS*, *atlA*, and *icaA*); the others are not directly associated with the *sarA *regulon (*arcA, spx*, and *tcaR*). Expression levels of these genes were analyzed in biofilm using the wild-type strain COL, the *msa *mutant, and the complemented *msa *mutant (Table 2). A change in expression level of twofold or higher was considered significant (Table 2).

Mutation of *msa *resulted in a significant increase in *alsS *expression in biofilm. The *alsSD *operon encodes acetolactate synthase and an acetolactate decarboxylase. Previous studies have reported that mutation of the *alsSD *operon in *S. aureus *resulted in a biofilm defect [17,18]. The biofilm defect of the *alsSD *mutant was attributed to the role of this operon in the production of acetoin from pyruvate [18,19]. Acetoin production is necessary for acid tolerance within biofilms [5,20]. The effect of over-expression of the *alsSD *operon as observed in the *msa S. aureus *mutant on biofilm formation is not clear. One might speculate that premature build up acetoin in the medium could signal exhaustion of glucose and lead to detachment of cells from biofilm. Further studies are needed to explore this possibility.

Mutation of *msa *resulted in a significant decrease in expression of *arcA *in biofilm (Table 2). The *arcA *gene encodes arginine deiminase which is a member of the arginine deaminase (ADI) pathway. This pathway is used to generate energy using arginine under anaerobic conditions, [21-23]. The results of several studies point to the importance of the ADI pathway in biofilm formation and pathogenesis. Some oral bacteria have been shown generate ammonia via the ADI pathway to maintain pH homeostasis in biofilms [24]. Other studies have shown that the ADI pathway was induced during biofilm formation [5]. Additionally, bacteria in biofilm selectively utilize six amino acids, including arginine [25], further demonstrating the importance of the ADI pathway in biofilm formation and pathogenesis and may explain the *msa *mutant biofilm phenotype. However, when *arcD *was disrupted in the *S. aureus *strain UAMS-1, the mutant formed effective biofilms and were as virulent as wild-type in a catheter infection mouse model despite the fact that PIA was significantly reduced [25]. These discrepant results may be due to differences in strains, growth conditions, or infection model but they clearly indicated the need for more studies in the role of arginine metabolism in biofilm development and pathogenesis.

Biofilm accumulation relies on cell-cell adhesion mediated by the polysaccharide intercellular adhesin (PIA), which is produced by the *icaADBC *operon, and was shown to play a major role in biofilm accumulation [26]. Recent studies, however, have indicated that the *icaADBC *operon is not essential for biofilm formation in some strains [5,15,27,28]. We studied the expression levels of genes encoded by this operon and found that the *msa *mutation reduced the expression of *icaA *in the post-exponential growth phase of planktonic cultures only (data not shown). In biofilm, however, the expression of *icaA *in the mutant was not significantly different from wild-type (Table 2). The *ica*-dependent pathway is primarily regulated by the *icaR *repressor [29]. When IcaR becomes activated by Spx, PIA levels are reduced [30,31]. We found that there was no significant difference in expression of *icaR *or *spx *in the *msa *mutant compared to the wild-type (data not shown). This is consistent with findings by Tu Quoc et al. [32] that some biofilm-defective mutants did not show altered PIA levels. Additionally, O'Neill *et al*. [7], recently showed that glucose-induced biofilm formation in MRSA strains is *ica*-independent. This is relevant to our data, since the COL strain is a MRSA strain and glucose was added to the culture media to induce biofilm in this study suggesting that *msa *is involved in biofilm formation using an *ica*-independent mechanism as was previously described in some strains [5,33,34]. Another important locus, *sasG*, which is similar to accumulation- associated protein in *S. epidermidis *has been shown to play a role in cell-cell adhesion and accumulation of biofilm [35,36], however, there was no change in expression of *sasG *between the *msa *mutant and wild type grown in planktonic cultures as determined by DNA microarray experiments in our lab (data not shown).

## Conclusion

In summary, mutation of the *msa *gene in strain COL of *S. aureus *results in a weak biofilm at the accumulation stage resulting in an immature biofilm. This defect is likely mediated by the reduced expression of *sarA *in the *msa *mutant. Our results suggested that the weak/unstable biofilm defect in the *msa *mutant is an intermediate phenotype between the *sarA *mutant and wild-type. However, we cannot rule out the contribution of other loci that fall under the influence of *msa *in a *sarA*-independent manner (e.g. *arcA*). Our findings emphasize the complex nature of biofilms and indicated that several independent regulators and environmental stimuli contribute to the establishment of sessile communities of *S. aureus*. The intermediate phenotype of the biofilm formed by the *msa *mutant is a helpful clue in deciphering the *sarA*-mediated mechanism of biofilm formation, which is still unclear. Sequence analysis shows that Msa is a putative membrane protein [8,37] suggesting that it may play a role in environmental sensing that feeds into the *sarA *regulon to contribute to biofilm formation.

## Methods

### Bacteria and growth conditions

The *S. aureus *biofilm-forming strain COL was used in this study. Strains were grown on tryptic soy agar (TSA) or in tryptic soy broth (TSB) at 37°C under constant aeration, supplemented with antibiotics where appropriate. Generalized transduction with phage Φ11 was used to generate an *msa *mutant and a complemented *msa *mutant in the COL strain, as described previously [8]. The media used in flow cells and microtitre plate assays was TSB supplemented with 3% sodium chloride and 0.5% glucose.

### Biofilm assays

Biofilm assays were performed in microtitre plates and flow cells, as described previously [4,5]. Briefly, flow cells (Stovall Life Science, Greensboro, NC) were pre-coated with human plasma. A suspension of bacteria from an overnight culture were then introduced into the flow cells by injection and allowed to incubate at 37°C for one hour. Media was then pumped through the flow cells at a flow rate of 0.5 ml/min. Flow cells were observed and photographed periodically. No antibiotic selection was used when growing the complemented *msa *mutant in biofilm.

### Binding assays

Host proteins binding assays were performed as we described previously [5]. These assays were used to compare the capacity of the *msa *mutant to bind fibrinogen and fibronectin.

### Initial adherence assay

We used two assays to measure the capacity of the *msa *mutant to bind surfaces with or without pre-coating with plasma. The first one was performed by modifying the microtitre biofim assay described previously [4]. Briefly, overnight cultures of *S. aureus *test strains were diluted to an OD^560 ^of 0.1 in fresh TSB, and 200 μl was added to each well (polystyrene pre-coated with human plasma) in triplicate. Following a 1 hour incubation at 37°C, the microtitre wells were washed three times with 1× PBS. Adherent cells were then fixed with 200 μl of 100% ethanol for 10 min. Ethanol was removed and the wells were air dried for 2 min. Adherent cells were stained for 2 minutes with 200 μl of 0.41% Crystal Violet (w/v in 12% ethanol), then washed three times with 1× PBS. Wells were allowed to dry, and then ethanol was used to elute the wells. Absorbance readings were taken at 595 nm using a Synergy2™ Multi-Mode Microplate Reader (BioTek Instruments, Inc. Winooski, Vermont). Data shown is the average of three independent experiments. The second assay measures adherence to a catheter without pre-coating with plasma. This assay was performed as previously described [38]. Briefly, staphylococcal strains were grown overnight in TSB and cultures were standardized to an OD_650 _of 0.1. Catheters (PE10; Becton Dickinson and Co., Sparks, MD.) were cut to a length of 0.5 cm and placed into appropriate cultures. After incubating the cultures at 37°C for 30 min, the catheters were removed using sterile forceps and washed five times in sterile PBS. After washing, the catheters were placed in 1% proteose peptone (Difco, Ann Arbor, MI.) and continuously vortexed for two minutes to release bacterial cells from the catheter. For bacterial enumeration, serial dilutions were plated onto TSA plates. The mean and standard errors were calculated for the adherence of each strain.

### Confocal microscopy

Confocal scanning laser microscopy was performed using a Carl-Zeiss LSM 510 META with excitation at 488 nm and emission collected at 500 to 530 nm (green channel). Image stacks of biofilms were taken from at least three distinct regions on the flow cell and were analyzed using the provided software (Carl-Zeiss, Inc., Peabody, Mass.). Thickness of the biofilm was measured starting from the z-section at the flow-cell/biofilm interface to the z-section at the top of the biofilm surface containing < 5% of total biomass.

### RNA isolation

Total RNA was isolated from *S. aureus *planktonic cultures using Qiagen RNeasy Maxi column (Qiagen) as previously described [8], based on a method developed by Lindsay and Foster [39]. *S. aureus *cultures were grown without antibiotic selection and under low-aeration conditions (150 r.p.m. at a media volume:flask volume ratio of 0.5). Cells were harvested at optical densities (OD_560_) of 0.3, 1.5 and 4.0, which correspond to the mid-exponential, late-exponential, and post-exponential growth phases, respectively. RNA from biofilms was accomplished by harvesting cells from flow cells as previously described [5] and using using Qiagen RNeasy Maxi column. The data shown is from three independent experiments.

### Real-time quantitative PCR

Gene expression analysis by real-time quantitative PCR (RT-qPCR) was performed as described previously [8], with all reactions done in triplicate. The constitutively expressed gyrase gene (*gyr*) was used as an endogenous control, as described previously [40]. Primer specificity and efficiency was measured as described previously [8]. Expression analysis for each gene was based on at least two independent experiments. Two-fold or higher changes in gene expression were considered significant.

## Authors' contributions

KS created the *msa *mutation, characterized the biofilm phenotype, analyzed gene expression and drafted the manuscript. AS carried out biofilm assays and analyzed gene expression. KS and AS contributed equally to this manuscript. VN analyzed sequence data of the *msa *mutant. MOE conceived of the study, participated in its design and coordination. All authors read and approved the final manuscript.
